# Network-level allosteric effects are elucidated by detailing how ligand-binding events modulate utilization of catalytic potentials

**DOI:** 10.1371/journal.pcbi.1006356

**Published:** 2018-08-07

**Authors:** James T. Yurkovich, Miguel A. Alcantar, Zachary B. Haiman, Bernhard O. Palsson

**Affiliations:** 1 Department of Bioengineering, University of California, San Diego, La Jolla, CA, USA; 2 Bioinformatics and Systems Biology Program, University of California, San Diego, La Jolla, CA, USA; 3 Department of Pediatrics, University of California, San Diego, La Jolla, CA, USA; Chalmers University of Technology, SWEDEN

## Abstract

Allosteric regulation has traditionally been described by mathematically-complex allosteric rate laws in the form of ratios of polynomials derived from the application of simplifying kinetic assumptions. Alternatively, an approach that explicitly describes all known ligand-binding events requires no simplifying assumptions while allowing for the computation of enzymatic states. Here, we employ such a modeling approach to examine the “catalytic potential” of an enzyme—an enzyme’s capacity to catalyze a biochemical reaction. The catalytic potential is the fundamental result of multiple ligand-binding events that represents a “tug of war” among the various regulators and substrates within the network. This formalism allows for the assessment of interacting allosteric enzymes and development of a network-level understanding of regulation. We first define the catalytic potential and use it to characterize the response of three key kinases (hexokinase, phosphofructokinase, and pyruvate kinase) in human red blood cell glycolysis to perturbations in ATP utilization. Next, we examine the sensitivity of the catalytic potential by using existing personalized models, finding that the catalytic potential allows for the identification of subtle but important differences in how individuals respond to such perturbations. Finally, we explore how the catalytic potential can help to elucidate how enzymes work in tandem to maintain a homeostatic state. Taken together, this work provides an interpretation and visualization of the dynamic interactions and network-level effects of interacting allosteric enzymes.

## Author summary

Enzymatic rate laws have historically been used to simulate the dynamics of complex metabolic networks with regulated reactions represented by allosteric rate laws. Here, we use detailed elementary reaction descriptions of regulatory enzymes that allow for the explicit computation of the fraction of the enzymes that are in a catalytically-active state. The fraction of the enzyme that is in the active state represents the time-dependent utilization of the enzyme’s “catalytic potential,” its capacity to catalyze a reaction. We apply this interpretation to red blood cell glycolysis, examining how three key kinases with allosteric regulation modulate their utilization of their catalytic potential based on ligand-binding events throughout the network in order to maintain a homeostatic state. We then examine how an enzyme modulates its utilization of its catalytic potential using personalized data as a case study, visualizing the systems-level properties of a kinetic model.

## Introduction

The human red blood cell (RBC) metabolic network has historically been the target of complex kinetic model building due to its relative simplicity and the vast amounts of data and information available on its biochemistry and physiology. RBCs lack cellular compartments (e.g., nuclei, mitochondria) and therefore certain cellular functions, such as transcriptional and translational regulation and the ability to use oxidative phosphorylation to produce energy [1]. As a result, glycolysis is the primary source of ATP generation for the RBC [2] and undergoes allosteric regulation at major control points. Glycolytic ATP production is thus largely regulated in response to the rate of ATP utilization of known cellular functions, mostly the ATP-driven sodium/potassium transmembrane pump.

Mathematical models have been used to study the dynamics of RBC metabolism since the 1970s [3]. Constraint-based modeling methods have been used to explore the mechanisms underlying cellular metabolism [4–6], and specialized methods have been developed that allow for the study of system dynamics [7–9]. Kinetic models represent an approach that has the potential to truly capture the temporal dynamics at short time scales [10–13]. The first whole-cell kinetic model of RBC metabolism was published in the late 1980s [14–17], with other such models produced since then [18–20]. More recently, so-called “enzyme modules” have been introduced and used to explicitly model detailed binding events of ligands involved in allosteric regulation as an alternative to the traditional use of allosteric rate laws [21, 22]. These enzyme modules provide a fine-grained view of the activity and state of a regulated enzyme. Further, they open up many new possibilities in understanding the metabolic regulation that results from complex interactions of regulatory signals, as well as providing a way to explicitly represent biological data types such as sequence variation and protein structures.

Historically, the primary way to visualize the output from a kinetic model is to plot the time profiles of individual network components (e.g., metabolite concentrations, enzymatic reaction rates). While these quantities are informative, they fail to provide insight into systemic qualities of the network. Dynamic phase portraits have been explored as an alternative [23]. With the formulation of enzyme modules, there is a need to study alternative ways to visualize network dynamics to bring about a new understanding of integrated functions similar to what Bode plots [24] or root loci [25] achieved in the early days of the development of classical control theory. Enzyme modules allow for the explicit computation of the fraction of the regulatory enzyme that is in an active state and generates the reaction flux. The collective action of all the ligands binding to the enzyme—through the computation of the active enzyme fraction—fundamentally represent its regulation.

In this study, we use previously described enzyme modules to model the allosteric regulatory effects of hexokinase (HEX), phosphofructokinase (PFK), and pyruvate kinase (PYK), the three major regulatory points in RBC glycolytic energy generation. We compute and visualize each kinase’s utilization of its catalytic potential as a function of the energy charge, a systemic variable. We analyze the response of each enzyme module to perturbations in ATP utilization, simulating the impact of various physiological stresses on the RBC that affect the energy charge (e.g., hypoxia). We then examine the robustness of the catalytic potential as a qualitative systemic measure of the state of an enzyme using randomized models and existing personalized data. Finally, we explore how the catalytic potential can be used to investigate how various enzymes work in tandem to respond to external perturbations.

### Defining the catalytic potential

**Fig 1:**
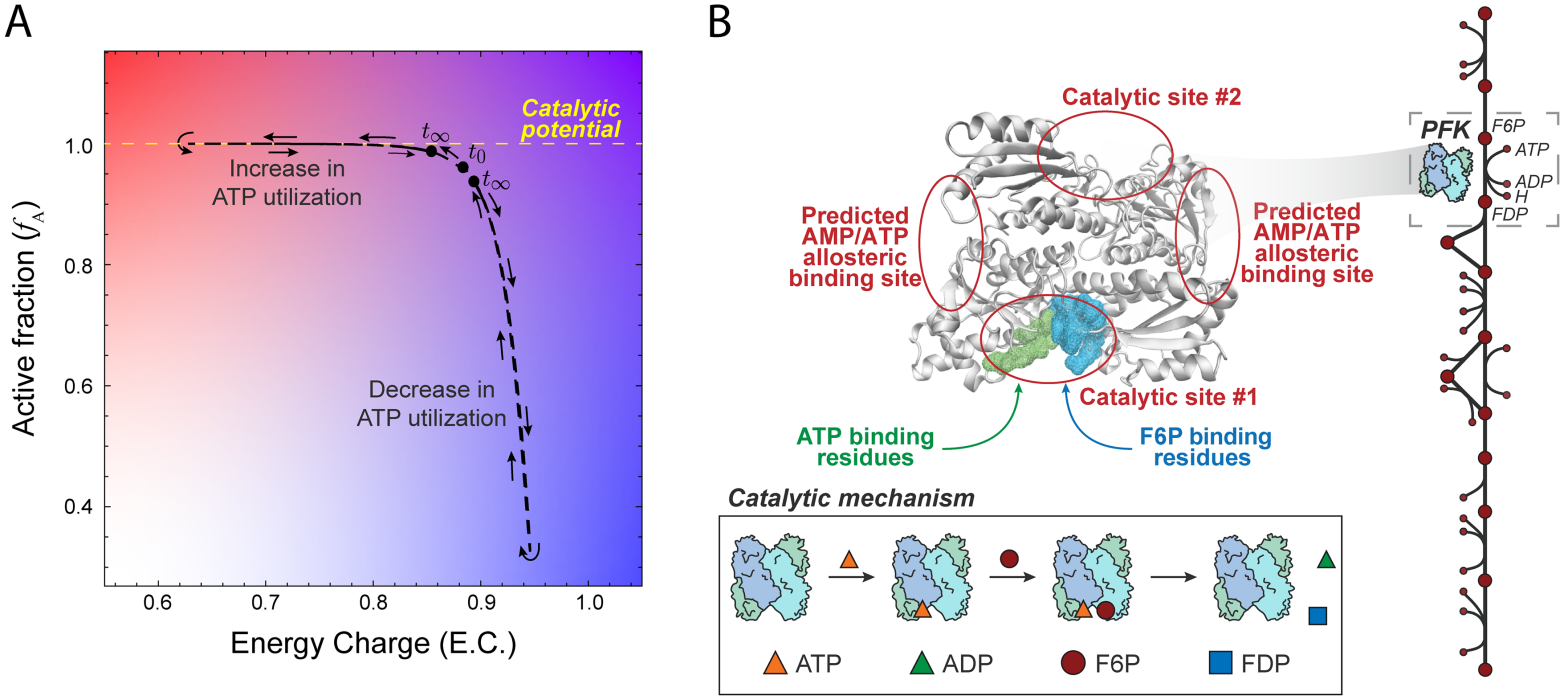
Definition of catalytic potential and modeling formalism. (A) The “catalytic potential” of an enzyme is its capacity to catalyze a reaction and can be visualized by computing the active fraction of enzyme (*f*_A_) as a function of the adenylate energy charge. Systemic perturbations (like adjusting the ATP utilization shown here) allow for a visualization of how an enzyme modulates its utilization of its catalytic potential in order to maintain a homeostatic state. (B) The structure of one of two PFK homomers along with the catalytic mechanism shows predicted allosteric binding sites for AMP and ATP [26]. Explicitly modeling elementary reaction steps and ligand-binding allows for the computation of the catalytically active enzyme fraction, *f*_A_.

We are interested in studying the “catalytic potential” of an enzyme—its capacity to catalyze a reaction—from a network-level perspective. An enzyme achieves its catalytic potential when all individual enzyme species are in an active form; an enzyme with allosteric regulation modulates its utilization of its catalytic potential based on ligand-binding events throughout the network in order to maintain a homeostatic state. Here, we propose that an enzyme’s utilization of its catalytic potential can be visualized by computing the fraction of total enzyme that is available to catalyze a reaction as a function of the adenylate energy charge (Fig 1A). In this section, we describe both of these properties and how they can be computed using enzyme modules and mass action kinetics.

The energetic state of a cell can be measured using the adenylate energy charge [27], which represents the amount of high energy bonds available in the adenosine phosphate pool. The energy charge is given by

$$\text{Energy}\;\text{Charge}=\frac{\left[\text{ATP}\right]+\frac{1}{2}\left[\text{ADP}\right]}{\left[\text{ATP}]+[\text{ADP}]+[\text{AMP}\right]}$$ (1)

where [AMP], [ADP], and [ATP] represent the concentrations of those respective metabolites. Because of the number of reactions in which the adenosine phosphates participate, the energy charge is a systemic variable sensed by important enzymatic regulators [28, 29] which can be more sensitive to perturbations than are reaction rates (Fig A in S1 File).

To examine individual enzymatic reactions that are regulated by at least one metabolite in the combined adenosine phosphate pool (i.e., AMP, ADP, or ATP) from a network-level perspective, we can compute properties of enzymes as a function of the energy charge. A kinetic model that explicitly represents each of the elementary steps for an enzymatic reaction (i.e., an enzyme module) provides enough detail to compute the fraction of uninhibited enzyme primed to facilitate the conversion of substrate to product for enzymes allosterically regulated through effector molecules. This catalytically active fraction (*f*_A_) can be calculated for an enzyme from

$$f_{\text{A}}=\frac{\sum_{i=0}^{n}R_{i}+R_{i,\text{A}}+R_{i,\text{AS}}}{E_{\text{total}}}$$ (2)

where *n* is the number of enzymatic binding sites, *R*_*i*_ is the unbound enzyme in the active state (i.e., not bound to inhibitors), *R*_*i*,A_ is the enzyme bound to the cofactor, *R*_*i*,AS_ is the enzyme bound to the substrate and cofactor, and *E*_total_ is the total amount of enzyme. The subscript *i* represents the amount of activators bound to allosteric sites; for tetrameric structures like PFK and PYK, *i* ranges between 0 and 4 [30, 31]. Here, we adopted the Monod-Wyman-Changeux (MWC) reaction framework [32] for PFK and PYK in which the allosteric activator and inhibitor can only bind to the relaxed and tense state, respectively. Both the energy charge and *f*_A_ were computed from model simulations.

We use mass action kinetics to model RBC glycolysis with enzyme modules (i.e., explicitly representing the elementary reactions for ligand-binding) for HEX, PFK, and PYK (see Supplementary Material for the full reaction mechanism for each enzyme module). In the following sections, we detail the construction and validation of models with enzyme modules and examine each enzyme’s utilization of its catalytic potential in response to perturbations in ATP utilization.

## Results

### Model construction and validation

We constructed a model of RBC metabolism that comprises glycolysis, the Rapoport-Luebering (RL) Shunt, and the interaction of hemoglobin with 2,3-diphosphoglycerate [23]; the stoichiometric matrix for the network, all kinetic parameters, and the initial flux values are provided in S1 Data. This small-scale model allows us to study the regulatory effects on glycolysis. This model contains three allosterically regulated kinases for which enzyme modules were constructed (see Methods and Supplementary Material): hexokinase (HEX), phosphofructokinase (PFK), and pyruvate kinase (PYK). To validate each of the enzyme modules, we sought to introduce physiologically relevant perturbations that would affect the energy charge. Several external pressures—such as hypoxic conditions [33] or sheer stress experienced *in vivo* due to arterial constriction [34]—can result in increased release of ATP from RBCs *in vivo*, while internal ATP concentrations can drop by as much as 27% or 50% due to aging or the presence of acute disease states such as gastrointestinal tumors [35]. To model these behaviors, we perturbed the rate of ATP utilization (see Methods) to induce a systemic response that is qualitatively representative of the observed phenotypes (increasing and decreasing the value of the rate constant for the hydrolysis of ATP; see Methods). We built and tested models with each enzyme module individually, examining its utilization of its catalytic potential of the enzyme as the system returns to its original homeostatic point. To validate these models against previous experimental results reported in the literature, we make the assumption that the initial velocity of a reaction is proportional to the amount of catalytically active enzyme, *f*_A_ [36]; the qualitative shape of a rate versus energy charge plot should then match that of an *f*_A_ versus energy charge plot.

#### Phosphofructokinase

**Fig 2:**
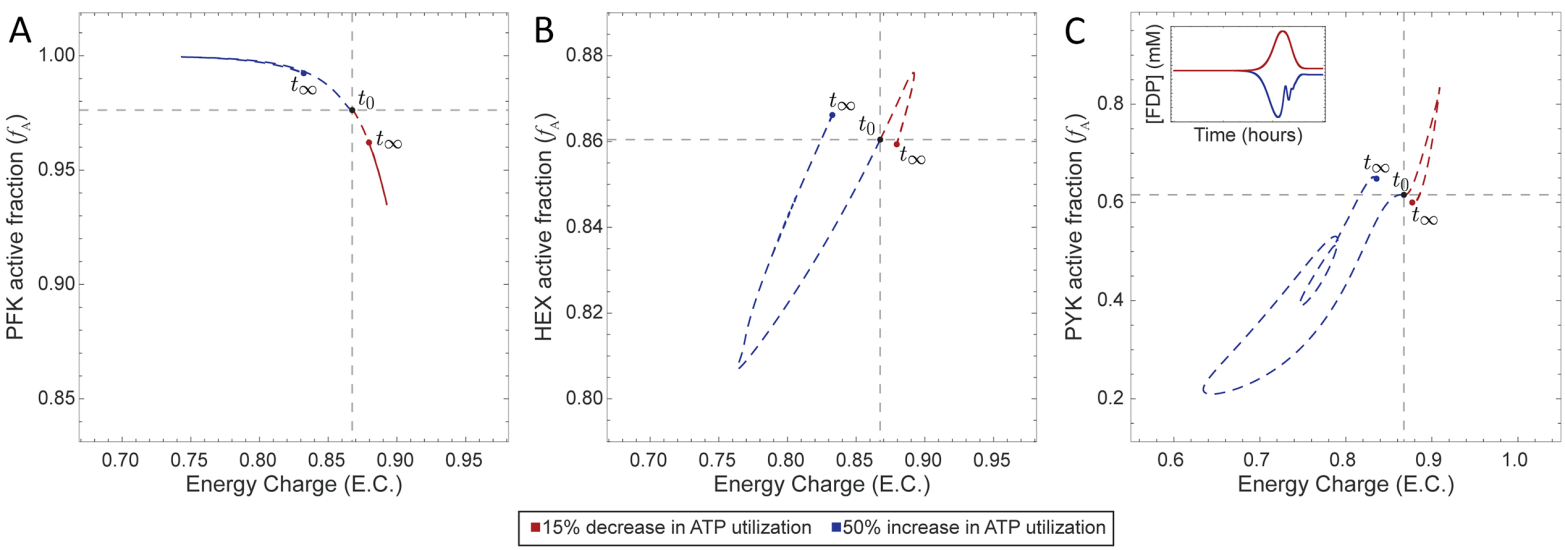
Catalytic potential plots for the base model (glycolysis, the RL Shunt, and hemoglobin) plus an enzyme module for (A) PFK, (B) HEX, and (C) PYK individually. The inset in panel (C) shows the concentration profile of FDP (see Fig D in S1 File for the detailed concentration profile).

Our primary test case was PFK, which plays a major role in determining glycolytic flux through the conversion of fructose 6-phosphate (F6P) to fructose 1,6-bisphosphate (FDP). The regulatory mechanism of PFK is complicated [37], so we here use a simplified reaction mechanism (Fig 1A) where PFK binds first to ATP, forming a complex that then binds F6P and then converts the two bound substrates to FDP producing ADP in the process (see Methods and Supplementary Material for full details). The four binding sites operate independently, i.e., they do not “cooperate.” The catalytic activity of PFK is controlled through allosteric regulation by AMP and ATP (Fig 1). AMP and ATP bind to an allosteric site distal to the catalytic site [26], inducing a conformational change that modulates the activity of PFK. We performed a dynamic simulation in which we perturbed the rate of ATP utilization, observing an inverse relationship between the catalytically active enzyme fraction and energy charge (Fig 2A); this result corroborated previously reported behavior for PFK [28]. We can see that PFK senses the change in energy charge and adjusts the flux through PFK to return the system to its original homeostatic state.

#### Hexokinase and pyruvate kinase

We also constructed enzyme modules for HEX and PYK, using mechanisms that allow the substrate to bind cofactors in any order (see Methods and Supplementary Material for full details). We inserted each of these enzymes modules into the base model (glycolysis, the RL Shunt, and hemoglobin) separately, resulting in a separate model for each enzyme module. We then performed the same perturbations (50% increase and 15% decrease in the rate of ATP utilization) and computed the energy charge and active fraction of each enzyme. We observed that the qualitative trends for HEX (Fig 2B) were in agreement with previously observed experimental evidence [38]. However, the behavior of the PYK module exhibited a direct relationship between *f*_A_ and energy charge (Fig 2C), an observation that conflicts with the inverse relationship previously observed *in vitro* [38].

### Robustness of the catalytic potential of an enzyme

The baseline RBC glycolytic model used to construct the models is based on nominal parameter values [23]. However, genetic variation in the human population leads to varying RBC metabolic dynamics in different individuals. Our next goal was therefore to explore the sensitivity of an enzyme’s catalytic potential to perturbations to model parameters. Because of its dependence on the energy charge and literature validation of its catalytic potential, PFK was used for an in depth exploration of the robustness of the catalytic potential.

We first generated 50 models from randomly sampled, thermodynamically feasible concentrations values (see Methods) and perturbed the rate of ATP utilization. We examined the net rate of ATP usage (i.e., total flux through ATP-producing reactions minus total flux through ATP-consuming reactions), the energy charge as a function of time, and the catalytic potential (Fig D in S1 File). From this analysis, we see that reaction rates (Fig E in S1 File) are not as sensitive to changes in ATP levels, while these changes are captured by the energy charge (Fig E in S1 File). The catalytic potential then allows us to incorporate this systemic information as we observe the response of PFK. However, while these randomized models were constructed with thermodynamically feasible metabolite concentrations, they do not necessarily represent physiologically feasible concentrations.

Therefore, we further collected previously reported RBC and plasma metabolite levels from a series of individuals [20], enabling the construction of “personalized” RBC models (see Methods). We constructed personalized models using glycolytic metabolite concentrations and equilibrium constants for nine individuals from a previous study [20]. Using personalized models provides a sensitivity analysis that examines physiologically-feasible parameter values.

**Fig 3:**
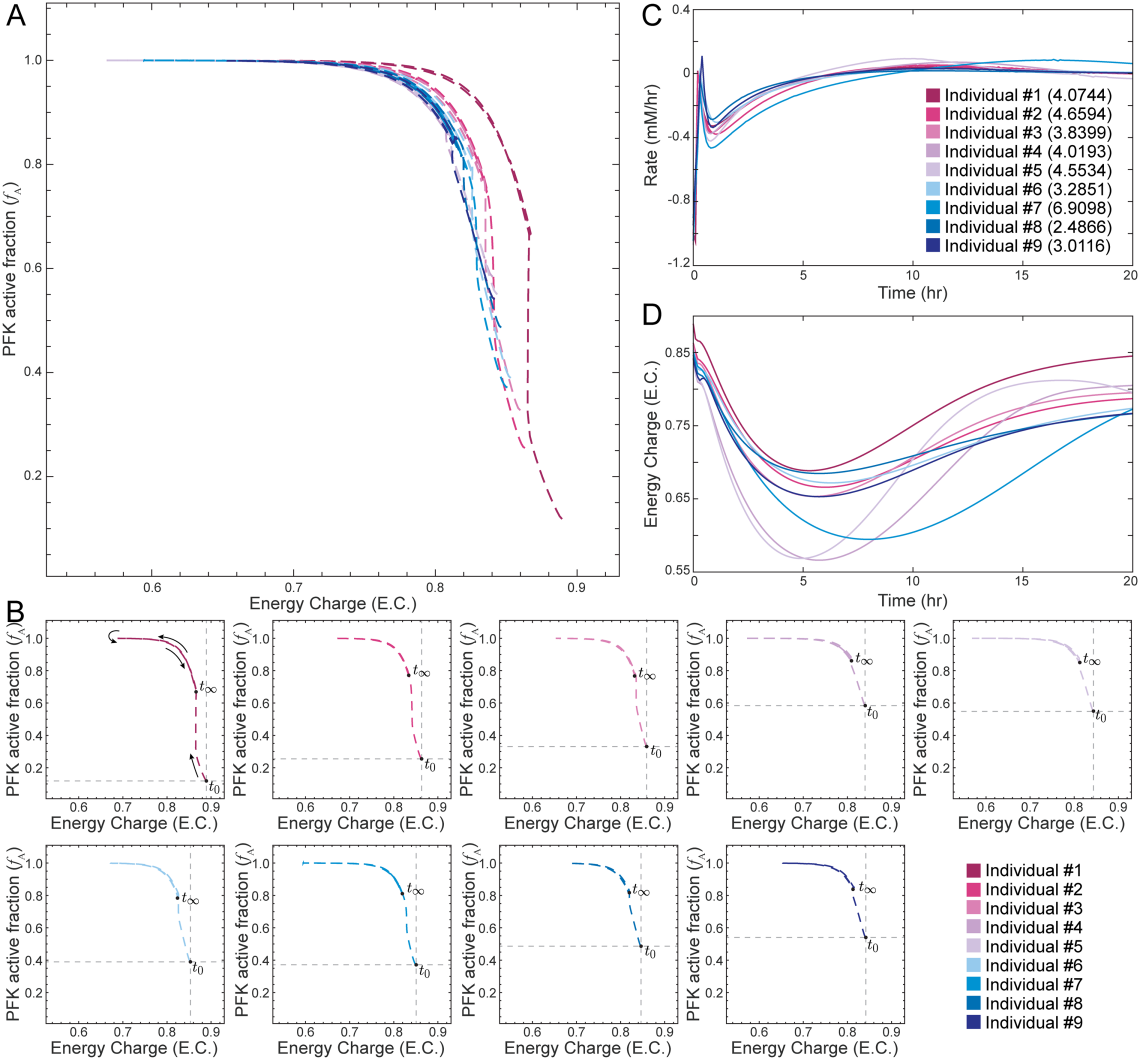
Disturbance rejection capabilities of personalized glycolytic models with an enzyme module for PFK and hemoglobin. (A) Superimposed catalytic potential plots for all personalized models. (B) Catalytic potential plots for each individual; the intersection of the gray lines denotes the initial steady-state value at time zero and helps show the differences among the population. (C) The net rate of ATP usage (i.e., total flux through ATP-producing reactions minus total flux through ATP-consuming reactions) is shown as a function of time. The number in parentheses represents the SSE for each model, quantifying the total deviation of the output from the setpoint. (D) The energy charge is shown as a function of time.

The general qualitative trend for the catalytic potential plot of PFK was similar to the one using literature values (Fig 2A), but initial *f*_A_ values were significantly lower in the personalized models (Fig 3A and 3B). In particular, the amount of active PFK for each individual reached a saturation point that was higher than the initial steady-state value in order to compensate for the increase in ATP utilization before returning to a final steady-state value. While we observe that there is little difference among the rate profiles (Fig 3C), we observe much greater differences in the catalytic potential plots (Fig 3A and 3B) and energy charge profiles (Fig 3D). Notably, the model for Individual #1 exhibited a much different response than the other eight personalized models (Fig 3A, 3B and 3D). We examined this behavior and determined that PFK is highly sensitive to the rate constants for the binding of ATP and F6P to PFK (outliers with over 99% confidence according to the Dixon’s *Q* test; see Methods for full details); these were the only rate constants that were deemed to be outliers out of all enzymatic reactions, showing that these rate constants are the parameters to which PFK is most sensitive.

### Interplay among enzymes

Finally, we examined how an enzyme’s utilization of its catalytic potential can be used to characterize the interplay among enzymes in the same model. We thus integrated the enzyme modules for all three kinases studied here (PFK, HEX, and PYK) into the base model and introduced the same ATP utilization perturbations. We examined the disturbance rejection capabilities of this complete model compared with models with fewer enzyme modules, noting increased regulation generally improved the ability of a model to maintain a homeostatic state (Fig A in S1 File) as expected [39–42]. The inclusion of multiple enzyme modules in the same model allows us to characterize how the three allosterically regulated enzymes interact in determining the system’s response to these perturbations through dynamic simulation.

**Fig 4:**
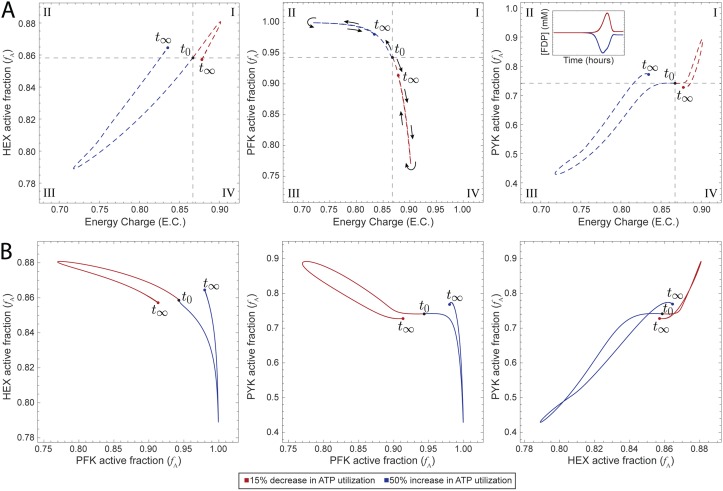
Dynamic responses of the base model with all three enzyme modules to perturbations in ATP utilization. (A) Catalytic potential plots for each of the enzyme modules as a function of the energy charge. Roman numerals indicate comparisons with the steady-state: (I) more enzyme in active form and higher energy charge; (II) more enzyme in active form and lower energy; (III) more enzyme in inactive form and lower energy charge; and (IV) more enzyme in inactive form and higher energy charge. The inset shows the concentration profile of FDP (see Fig D in S1 File for the detailed concentration profile). (B) Phase portraits displaying pairwise relationships between the active fractions of two kinases.

We characterized the catalytic potential of this complete model’s response to external perturbations (Fig 4A). We observed similar qualitative responses for each of the enzymes in the combined model as for each enzyme module individually (Fig 2). To examine the interplay between enzymes, we looked at phase portraits comparing the catalytically active enzyme fraction (*f*_A_) for each pairwise combination of enzymes (Fig 4B and S1 Video). We can see that as a greater fraction of PFK entered a more catalytically active state, a greater fraction of HEX become catalytically inactive; a similar behavior was observed for the PFK-PYK pair. We observed that HEX and PYK moved in tandem, with both enzymes moving into catalytically active or inactive states together. This behavior is likely due to the fact that these enzymes represent the boundaries of the system and therefore are linked in order to maintain system stability.

## Discussion

The ability to mechanistically model cellular metabolism allows for the construction of predictive physiological models. However, the mechanistic results obtained from time-course plots can complicate the interpretation and analysis of systems-wide responses to relevant perturbations. To help provide a better method of elucidating this behavior, we built modularized glycolytic models with enzymes serving as regulators that allows for a new interpretation of the state of an enzyme—where it operates with respect to its maximum catalytic potential. These models were then validated against existing empirical data to understand the relationship between the catalytically active enzyme fraction and energy charge. Visualizing an enzyme’s utilization of its catalytic potential allowed for the analysis of important systems behaviors. The results presented here have two primary implications.

First, we have studied glycolysis from a perspective in which enzymes are regulators. Individual kinases serve as tuning dials for the system by sensing changes in energy charge and modulating their utilization of their catalytic potentials in order to return the system to a homeostatic state. If the energy charge dropped, then mass action kinetics would dictate that more flux would be pushed through a reaction that produces ATP in order to increase the energy charge. The response of PFK showed that its regulation is strong enough to overcome the dynamics that would result from these mass action trends alone. HEX behaves as is expected due to mass action (a lower energy charge results in a reduced fraction of catalytically active enzyme), but the observed behavior of PYK is opposite what would be expected based on the law of mass action. A decrease in energy charge would intuitively result in more catalytically active PYK since that would then result in more ATP. The literature reports this expected behavior for initial velocity of PYK [38]. However, these assays did not contain FDP, an allosteric activator of PYK. We observed that an increase in energy charge led to an initial increase in FDP concentration and a corresponding increase in the amount of PYK in the catalytically active form (Figs 2C and 4A). These plots suggest that the regulation of PYK by FDP leads to this unintuitive behavior.

Second, we have shown that examining an enzyme’s catalytic potential can provide additional insight into how metabolic networks maintain a homeostatic state following physiologically-relevant perturbations. A small-scale model that explicitly accounted for the regulatory mechanisms of the three glycolytic kinases allowed us to directly investigate the interplay among these three enzymes (S1 Video). When we applied this metric to examine the response of personalized models to ATP utilization perturbations, we observed differences that were not apparent simply from the rate profile. The kinases modulated the response of the system, as demonstrated by examining individual parameterization of personalized models (Fig 3). Through an examination of how PFK operates with respect to its catalytic potential, we were able to gain insight into how the regulator within a model is tuned in different individuals in order to maintain homeostasis (Fig 3A, 3B and 3D), a behavior that was not discernible through more typical metrics like rates of reaction (Fig 3C). Hence, the catalytic potential plots describe how enzymatic entities respond to system-wide changes in order to drive the cell towards a homeostatic state after environmental alterations. Upon further investigation, we determined that the utilization of catalytic potential for Individual #1 was different than the others due to differences in the binding affinities of ATP and F6P to PFK, indicating that the PFK module was most sensitive to these parameters. Thus, the catalytic potential helped provide insight into how subtle differences among individuals can lead to differing systemic responses to perturbations that push the system away from the homeostatic state.

The use of kinetic models to study the dynamics of cellular metabolism presents many well-documented challenges and limitations [43, 44]. Many of these issues revolve around attempting to parameterize biochemical processes that may not be well understood [43], one of the reasons that we adopted several simplified approaches in this study. Here, we employed the use of so-called “enzyme modules” (explicit representations of all ligand-binding reactions [21, 22]) for the allosterically regulated kinases in glycolysis, a modeling formulation which allowed us to compute the catalytically active enzyme fraction. We used the same reaction mechanisms (predicted by a computational method) from the previous study using enzyme modules [22] because our focus here was on interpreting the output from enzyme modules. Many alternative mechanisms exist [45], and the impact of employing different mechanisms on computing *f*_A_ could be explored in the future. Mass action kinetics were used for the other enzymes in the network and represent an approximation previously examined in the literature [22]. While the final reaction step for each enzyme module could be represented by two bimolecular steps [46], we have used a simplified termolecular step (i.e., all bound molecules are released in a single reaction step) due to a lack of high-confidence kinetic parameters. Kinetic models of metabolism are generally stiff systems [12], and the inclusion of enzyme modules exacerbates this issue due to the addition of several reactions with concentration variables that span several orders of magnitude (PFK module: 24 reactions; HEX module: 8 reactions; PYK: 34 reactions; see Supplementary Material for full mechanisms). Finally, the size of a model inevitably impacts the behavior of a model; we have chosen to draw our system boundary at the end of glycolysis, thereby not accounting for any downstream effects on the activity of PYK (such as flux leaving the pyruvate node and entering the citric acid cycle remnant reactions).

The RBC metabolic network consists of well-studied metabolic pathways and their associated metabolites. New methods for the visualization of regulatory behaviors—such as the catalytic potential plot introduced here—can lead to new insights and discoveries. We have evaluated the utilization of an enzyme’s catalytic potential as a sensor which can be used to visualize the state of that enzyme in the context of the metabolic network. Viewing enzymes as regulators through which we can tune the system response opens the door for us to investigate what the optimal state might be and how that state helps maintain homeostasis.

## Methods

All calculations were performed in Mathematica 11.1 [47]. Simulations were conducted using the Mass Action Stoichiometric Simulation (MASS) Toolbox kinetic modeling package (https://github.com/opencobra/MASS-Toolbox). Details for formulating a MASS model are found in Jamshidi et al. [21]. The system of ordinary differential equations was solved using the built-in Mathematica solver, which is embedded within the MASS Toolbox. All models used are provided in S2 Data.

### Glycolysis and the Rapoport-Luebering Shunt

The base glycolysis network included all 10 glycolytic enzymes and lactate dehydrogenase; the complete stoichiometric matrix is provided in S1 Data. Reaction rates were defined using mass action kinetics, representing enzyme catalysis as a single step. These simplified reactions were systematically replaced with enzyme modules following the procedure outlined by Du et al. [22]. Additionally, a phosphate exchange reaction was incorporated into the glycolytic network utilizing parameters obtained from Prankerd et al. [48]. Similarly, the Rapoport-Luebering Shunt was included in some models to account for the presence of hemoglobin, whose binding to oxygen is regulated by 2,3-diphosphoglycerate (2,3-DPG). Incorporation of this shunt was accompanied by parameter changes as previously described [23]. All model parameters are provided in S1 Data.

#### Comparison against cell-scale model

We compared our model against the cell-scale model constructed by Bordbar et al. [20]. Our model is based on this larger model and comprises a subset of the metabolic network (described above). We compared the qualitative behavior of our small-scale model against that of the full cell-scale model in response to a pulse that increased the ATP concentration by 50% and observed similar qualitative responses for the fluxes through each of the studied kinases and the energy charge (Fig F in S1 File).

### Enzyme module construction

Regulation was manually incorporated into the enzyme reactions. Initial conditions from the glycolysis and hemoglobin MASS toolbox example data were used in conjunction with equilibrium constants which were obtained from from various sources (see Supplementary Material). These values were subsequently utilized to solve for new kinetic parameters by setting the following constraint:

$$\frac{d\vec{x}}{dt}=S\cdot v\left(x;k\right)=0$$ (3)

where $d\vec{x}/dt$ is the concentration rate of change with respect to time for metabolites, **S** is the stoichiometric matrix, and **v(x**; **k)** is a vector containing reaction fluxes as a function of metabolite concentrations (**x**) and rate constants (**k**).

The parameters for all enzyme modules were determined using the methods described by Du et al. [22]. In short, the workflow includes: (1) defining all ligand-binding events and their associated equilibrium constants, (2) symbolically solving the resultant steady state mass balance, (3) solving for the pseudo-first-order elementary rate constant (kPERC) [23] of each enzymatic reaction using the overall flux state as a constraint, and (4) using the estimated kPERCs to approximate steady state concentration values for each enzyme form (e.g., enzyme bound to all combinations of ligands). The kPERC for a reaction is estimated using the following equation:

$$k_{i}=\frac{v_{i}}{\Pi_{i}{\text{reactants}}_{i}-\Pi_{i}{\text{products}}_{i}/K_{\text{eq}}}$$ (4)

where *k*_*i*_ is the kPERC for reaction *i* and *v*_*i*_ is the flux through that reaction [23]; reactions assumed to be irreversible were assigned an arbitrarily high *K* − _eq_ (Mathematica allows for the assignment of infinity).

We constructed a total of ten different models with varying amounts of regulation, spanning from the base glycolytic model with no enzyme modules (and therefore no regulation) to a model with three enzyme modules and the Rapaport-Luebering Shunt. The remaining models represented each combination of the three kinase modules. Enzyme module incorporation was accompanied by the deletion of the original single-step reaction in order to avoid redundant reactions. Stability for all systems was verified by simulating the network and ensuring that a steady-state point was found for all metabolites.

#### Hexokinase (HEX)

HEX (EC 2.7.1.1) was modeled as a monomer to account for the fact that it contains only one active catalytic site. The previously specified mechanism was chosen to match that used by [22] because all kinetic parameters were obtained from this source. A hemoglobin module is necessary to include when the HEX module is included because it affects the level of 2,3-DPG, which serves as a regulatory molecule for HEX. The full mechanism used for the HEX module is provided in the Supplementary Material.

#### Phosphofructokinase (PFK)

PFK (EC 2.7.1.11) was modeled as a homotetramer to account for its four catalytic and allosteric binding sites [49]. The previously specified mechanism was chosen to match that used by [22] because all kinetic parameters were obtained from this source; this mechanism does not account for cooperative binding. The full mechanism used for the PFK module is provided in the Supplementary Material.

#### Pyruvate kinase (PYK)

PYK (EC 2.7.1.40) was modeled to include allosteric regulation. Additional reactions were also included to account for the equilibration of both enzymes between the relaxed (R) and tense (T) state [31]. Additionally, PYK was modeled as a tetramer to account for the four catalytic and allosteric sites on each enzyme. Dissociation constants were obtained from [16] and rate constants were solved using Eq (3). The full mechanism used for the PYK module is provided in the Supplementary Material.

### Simulating ATP utilization perturbations

In order to mimic a physiologically-relevant perturbation away from the homeostatic state, we simulated a 50% increase in ATP utilization for 1,000 hours and a 15% decrease in ATP utilization [33–35]. These magnitudes were chosen because they resulted in observable changes in the energy charge which could then be used to qualitatively assess the impact on the system. Changes in ATP utilization were applied by changing the rate (*k*_ATP_) associated with ATP hydrolysis:

$$\begin{array}{c} \text{ATP}+{\text{H}}_{2}\text{O}\overset{k_{\text{ATP}}}{⇌}\text{ADP}+\text{H}+{\text{P}}_{\text{i}} \end{array}$$ (5)

where P_i_ represents inorganic phosphate which was modeled as a variable quantity to allow the system to respond to these perturbations. Increasing this rate decreases the amount of available ATP and ADP. We calculated the sum of squared error (SSE) for each model in order to quantify the total deviation of the output from its setpoint, which is zero. The resulting quantity (i.e., the SSE) is compared between models, with a smaller value indicating better disturbance rejection capabilities.

### Sensitivity analysis

#### Randomized models

Using the procedure outlined in Du et al. [22], we constructed 50 randomized models based on thermodynamically feasible metabolite concentrations that comprised glycolysis, the RL Shunt with hemoglobin, and PFK. In short, we utilized the cell-scale model constructed by Bordbar et al. [20] for parameter sampling, with the concentration range based on the measurements from that study. Thermodynamically feasible metabolite concentrations were then generated using equilibrium constants derived from eQuilibrator [50] and sampled using the COBRA Toolbox [51]. kPERCs were then calculated from these parameters and fluxes from the base model as described above, resulting in 50 randomized models.

#### Personalized models

Personalized models were constructed by replacing all primary intracellular glycolytic metabolite concentrations and equilibrium constants with values reported by Bordbar et al. [20]. New pseudo-elementary rate constant (PERC) values were calculated using the personalized concentration data as initial conditions instead of the nomial values used to formulate the non-personalized models. The PFK enzyme modules was parameterized for all individuals using the resulting concentration values after the addition of the Rapoport-Luebering pathway. The models used in the original publication accounted for a much larger network than just glycolysis (our focus here), resulting in potentially infeasible parameter sets. We encountered numerical issues due to the stiffness of the system, and thus we only used 9/24 of the models available in [20]; these data and model parameters are provided in S1 Data. Individuals #1-9 in our study correspond to individuals 2, 4, 5, 6, 7, 8, 10, 16, and 18, respectively, from [20].

To identify outliers within the reaction PERCs compared with the other personalized models, we performed a Dixon’s *Q* test [52]:

$$Q=\frac{\text{gap}}{\text{range}}$$ (6)

where the gap is the absolute difference between the point in question and the nearest value, and the range is the range of all values. For a set with nine samples, we can be 99% confident that a point is an outlier if the *Q* value is greater than 0.598; the *Q* values for the ATP and F6P binding steps had *Q* values of 0.84257 and 0.73164, respectively.

### System analysis

Rate pools for enzymes were defined as the rate at which enzyme produced product. This was accomplished by defining a pool from the product’s ODE consisting solely of the terms contributing to product formation. In other words:

$${\text{rate}}_{\text{enzyme}}=\sum v_{\text{formation}}$$ (7)

where *v*_formation_ represents the forward rate of the enzyme reaction and possesses units of mmol/L ⋅ hr. Defining the rate pools in this manner neglected effects of reversible reactions contributing to the formation of product. A negative value corresponds to a net-consumption of ATP.

## Supporting information

S1 File Supplementary text and figures. (PDF)

S1 Data Parameter values for all models and model structure. (A) Parameterization for non-personalized models. (B) Initial flux values for non-personalized models. (C) Parameterization for personalized models. (D) Initial flux values for personalized models. (E) Stoichiometric (**S**) matrix for system. (F) Metabolite list and metabolite ID mapping for **S** matrix. (G) Reaction list and reaction ID mapping for **S** matrix.
(XLSX)

S2 Data Zipped archive containing Mathematica model files and an example Mathematica notebook for calculating an enzyme’s utilization of its catalytic potential. (ZIP)

S1 Video Animation of 3-dimensional phase portraits for a model with all three kinase modules. (MP4)
