# Supplementary material for: Network-level allosteric effects are elucidated by detailing how ligand-binding events modulate utilization of catalytic potentials
Source: PLoS Comput Biol. 2018 Aug 7;14(8):e1006356. doi: 10.1371/journal.pcbi.1006356 (PMC6097697; doi:10.1371/journal.pcbi.1006356)
Supplement: S1 File — (PDF) [file pcbi.1006356.s001.pdf]

# Supporting Information for "Network-level allosteric effects are elucidated by detailing how ligand-binding events modulate utilization of catalytic potentials"

**JT Yurkovich et al.**

**PFK mechanism.** Phosphofructokinase (PFK, EC 2.7.1.11) was modeled as a tetrameric enzyme. The reaction mechanism for PFK includes allosteric activation and inhibition by AMP and ATP, respectively, where these regulators are capable of binding four sites distal to the active sites. Since the reaction catalyzed by PFK is essentially irreversible (1), the final catalytic reaction step was defined as irreversible. The overall reaction schema was chosen to match those used by Du et al. and Palsson (2, 3). Although the final termolecular reactions could be described as two separate bimolecular steps (4), the catalytic mechanisms are simplified such that the enzyme releases all bound metabolites upon catalysis, a simplification applied to all enzyme modules used throughout this study that eliminates the need for additional parameterization of potentially unknown values (e.g., equilibrium constants for each reaction).

We have here used the notation $\mathbf{PFK_i^*}$ to denote the relaxed (i.e., active) form $i$ of PFK and the notation $\mathbf{PFK_i^\dagger}$ to denote the tense (i.e., inactive) form $i$ of PFK; $i$ indicates the number of allosteric activators (AMP) or inhibitors (ATP) bound to the enzyme. The Monod-Wyman-Changeux (MWC) reaction framework (5) was adopted for PFK, wherein the allosteric activator and inhibitor can only bind to the relaxed and tense state, respectively. The full reaction schema is detailed in Eqs. (1)-(24):

$$\text{ATP} + \mathbf{PFK_0^*} \rightleftharpoons \mathbf{PFK_0^*}\text{-ATP} \tag{1}$$
$$\text{F6P} + \mathbf{PFK_0^*}\text{-ATP} \rightleftharpoons \mathbf{PFK_0^*}\text{-ATP-F6P} \tag{2}$$
$$\mathbf{PFK_0^*}\text{-ATP-F6P} \rightarrow \text{ADP} + \text{FDP} + \text{H} + \mathbf{PFK_0^*} \tag{3}$$
$$\text{AMP} + \mathbf{PFK_0^*} \rightleftharpoons \mathbf{PFK_1^*} \tag{4}$$
$$\text{ATP} + \mathbf{PFK_1^*} \rightleftharpoons \mathbf{PFK_1^*}\text{-ATP} \tag{5}$$
$$\text{FDP} + \mathbf{PFK_1^*}\text{-ATP} \rightleftharpoons \mathbf{PFK_1^*}\text{-ATP-F6P} \tag{6}$$
$$\mathbf{PFK_1^*}\text{-ATP-F6P} \rightarrow \text{ADP} + \text{FDP} + \text{H} + \mathbf{PFK_1^*} \tag{7}$$
$$\text{AMP} + \mathbf{PFK_1^*} \rightleftharpoons \mathbf{PFK_2^*} \tag{8}$$
$$\text{ATP} + \mathbf{PFK_2^*} \rightleftharpoons \mathbf{PFK_2^*}\text{-ATP} \tag{9}$$
$$\text{F6P} + \mathbf{PFK_2^*}\text{-ATP} \rightleftharpoons \mathbf{PFK_2^*}\text{-ATP-F6P} \tag{10}$$
$$\mathbf{PFK_2^*}\text{-ATP-F6P} \rightarrow \text{ADP} + \text{FDP} + \text{H} + \mathbf{PFK_2^*} \tag{11}$$
$$\text{AMP} + \mathbf{PFK_2^*} \rightleftharpoons \mathbf{PFK_3^*} \tag{12}$$
$$\text{ATP} + \mathbf{PFK_3^*} \rightleftharpoons \mathbf{PFK_3^*}\text{-ATP} \tag{13}$$
$$\text{F6P} + \mathbf{PFK_3^*}\text{-ATP} \rightleftharpoons \mathbf{PFK_3^*}\text{-ATP-F6P} \tag{14}$$
$$\mathbf{PFK_3^*}\text{-ATP-F6P} \rightarrow \text{ADP} + \text{FDP} + \text{H} + \mathbf{PFK_3^*} \tag{15}$$
$$\text{AMP} + \mathbf{PFK_3^*} \rightleftharpoons \mathbf{PFK_4^*} \tag{16}$$
$$\text{ATP} + \mathbf{PFK_4^*} \rightleftharpoons \mathbf{PFK_4^*}\text{-ATP} \tag{17}$$
$$\text{F6P} + \mathbf{PFK_4^*}\text{-ATP} \rightleftharpoons \mathbf{PFK_4^*}\text{-ATP-F6P} \tag{18}$$
$$\mathbf{PFK_4^*}\text{-ATP-F6P} \rightarrow \text{ADP} + \text{FDP} + \text{H} + \mathbf{PFK_4^*} \tag{19}$$
$$\mathbf{PFK_0^*} \rightleftharpoons \mathbf{PFK_0^\dagger} \tag{20}$$
$$\text{ATP} + \mathbf{PFK_0^\dagger} \rightleftharpoons \mathbf{PFK_1^\dagger} \tag{21}$$
$$\text{ATP} + \mathbf{PFK_1^\dagger} \rightleftharpoons \mathbf{PFK_2^\dagger} \tag{22}$$
$$\text{ATP} + \mathbf{PFK_2^\dagger} \rightleftharpoons \mathbf{PFK_3^\dagger} \tag{23}$$
$$\text{ATP} + \mathbf{PFK_3^\dagger} \rightleftharpoons \mathbf{PFK_4^\dagger} \tag{24}$$

The active fraction for PFK ($f_\text{A}^\text{PFK}$) is given by the summation of all active forms of the enzyme ($\mathbf{PFK_i^*}$ and bound species) over the total enzyme, $E_\text{T}$, given by:

$$f_\text{A}^\text{PFK} = \frac{\lambda}{\text{E}_\text{T}} \tag{25}$$

where

$$\lambda = \mathbf{PFK_0^*} + \mathbf{PFK_1^*} + \mathbf{PFK_2^*} + \mathbf{PFK_3^*} + \mathbf{PFK_4^*} + \mathbf{PFK_0^*}\text{-ATP} + \mathbf{PFK_1^*}\text{-ATP} + \mathbf{PFK_2^*}\text{-ATP} + \mathbf{PFK_3^*}\text{-ATP} + \mathbf{PFK_4^*}\text{-ATP} + \mathbf{PFK_0^*}\text{-ATP-F6P} + \mathbf{PFK_1^*}\text{-ATP-F6P} + \mathbf{PFK_2^*}\text{-ATP-F6P} + \mathbf{PFK_3^*}\text{-ATP-F6P} + \mathbf{PFK_4^*}\text{-ATP-F6P}.$$

**HEX mechanism.** Hexokinase (HEX) was modeled as a monomeric enzyme with a single active site. Inhibition was carried out by ADP, 2,3-DPG, and G6P (6–8). We have here used the notation $\mathbf{HEX}^*$ to denote the relaxed (i.e., active) forms of HEX and the notation $\mathbf{HEX}^\dagger$ to denote the tense (i.e., inactive) forms of HEX. The reaction mechanism mimicked that used by Du et al. (2) and is detailed in Eqs. (26)-(33):

$$\mathbf{HEX}^* + \text{ATP} \rightleftharpoons \mathbf{HEX}^*\text{-ATP} \tag{26}$$
$$\mathbf{HEX}^* + \text{GLC} \rightleftharpoons \mathbf{HEX}^*\text{-GLC} \tag{27}$$
$$\mathbf{HEX}^* + \text{G6P} \rightleftharpoons \mathbf{HEX}^\dagger\text{-G6P} \tag{28}$$
$$\mathbf{HEX}^* + \text{ADP} \rightleftharpoons \mathbf{HEX}^\dagger\text{-ADP} \tag{29}$$
$$\mathbf{HEX}^*\text{-ATP} + \text{GLC} \rightleftharpoons \mathbf{HEX}^*\text{-ATP-GLC} \tag{30}$$
$$\mathbf{HEX}^*\text{-GLC} + \text{ATP} \rightleftharpoons \mathbf{HEX}^*\text{-ATP-GLC} \tag{31}$$
$$\mathbf{HEX}^*\text{-GLC} + \text{23DPG} \rightleftharpoons \mathbf{HEX}^\dagger\text{-GLC-23DPG} \tag{32}$$
$$\mathbf{HEX}^*\text{-ATP-GLC} \rightleftharpoons \mathbf{HEX}^* + \text{ATP} + \text{GLC} \tag{33}$$

The active fraction for HEX ($f_\text{A}^\text{HEX}$) is given by the summation of all active forms of the enzyme ($\mathbf{HEX}^*$ and bound species) over the total enzyme, $E_\text{T}$, given by:

$$f_\text{A}^\text{HEX} = \frac{\lambda}{\text{E}_\text{T}} \tag{34}$$

where

$$\lambda = \mathbf{HEX}^* + \mathbf{HEX}^*\text{-ATP} + \mathbf{HEX}^*\text{-GLC} + \mathbf{HEX}^*\text{-ATP-GLC}.$$

**PYK mechanism.** Pyruvate kinase (PYK) was modeled as a tetrameric enzyme with allosteric activation by FDP and allosteric inhibition by ATP. This reaction mechanism allows these regulators to bind to four sites distal to the active sites. Because the reaction catalyzed by PYK is very close to irreversible (9), we have here modeled the last catalytic reaction to be irreversible.

We have here used the notation $\mathbf{PYK_i^*}$ to denote the relaxed (i.e., active) form $i$ of PYK and the notation $\mathbf{PYK_i^\dagger}$ to denote the tense (i.e., inactive) form $i$ of PYK; $i$ indicates the number of allosteric activators (FDP) or inhibitors (ATP) bound to the enzyme. The Monod-Wyman-Changeux (MWC) reaction framework (5) was adopted for PYK, wherein the allosteric activator and inhibitor can only bind to the relaxed and tense state, respectively. The full reaction schema is detailed in Eqs. (35)-(68):

$$\text{ADP} + \mathbf{PYK_0^*} \rightleftharpoons \mathbf{PYK_0^*}\text{-ADP} \tag{35}$$
$$\text{PEP} + \mathbf{PYK_0^*} \rightleftharpoons \mathbf{PYK_0^*}\text{-PEP} \tag{36}$$
$$\text{PEP} + \mathbf{PYK_0^*}\text{-ADP} \rightleftharpoons \mathbf{PYK_0^*}\text{-ADP-PEP} \tag{37}$$
$$\text{ADP} + \mathbf{PYK_0^*}\text{-PEP} \rightleftharpoons \mathbf{PYK_0^*}\text{-ADP-PEP} \tag{38}$$
$$\mathbf{PYK_0^*}\text{-ADP-PEP} + \text{H} \rightarrow \text{ATP} + \text{PYR} + \mathbf{PYK_0^*} \tag{39}$$
$$\text{FDP} + \mathbf{PYK_0^*} \rightleftharpoons \mathbf{PYK_1^*} \tag{40}$$
$$\text{ADP} + \mathbf{PYK_1^*} \rightleftharpoons \mathbf{PYK_1^*}\text{-ADP} \tag{41}$$
$$\text{PEP} + \mathbf{PYK_1^*} \rightleftharpoons \mathbf{PYK_1^*}\text{-PEP} \tag{42}$$
$$\text{PEP} + \mathbf{PYK_1^*}\text{-ADP} \rightleftharpoons \mathbf{PYK_1^*}\text{-ADP-PEP} \tag{43}$$
$$\text{ADP} + \mathbf{PYK_1^*}\text{-PEP} \rightleftharpoons \mathbf{PYK_1^*}\text{-ADP-PEP} \tag{44}$$
$$\mathbf{PYK_1^*}\text{-ADP-PEP} + \text{H} \rightarrow \text{ATP} + \text{PYR} + \mathbf{PYK_1^*} \tag{45}$$
$$\text{FDP} + \mathbf{PYK_1^*} \rightleftharpoons \mathbf{PYK_2^*} \tag{46}$$
$$\text{ADP} + \mathbf{PYK_2^*} \rightleftharpoons \mathbf{PYK_2^*}\text{-ADP} \tag{47}$$
$$\text{PEP} + \mathbf{PYK_2^*} \rightleftharpoons \mathbf{PYK_2^*}\text{-PEP} \tag{48}$$
$$\text{PEP} + \mathbf{PYK_2^*}\text{-ADP} \rightleftharpoons \mathbf{PYK_2^*}\text{-ADP-PEP} \tag{49}$$
$$\text{ADP} + \mathbf{PYK_2^*}\text{-PEP} \rightleftharpoons \mathbf{PYK_2^*}\text{-ADP-PEP} \tag{50}$$
$$\mathbf{PYK_2^*}\text{-ADP-PEP} + \text{H} \rightarrow \text{ATP} + \text{PYR} + \mathbf{PYK_2^*} \tag{51}$$
$$\text{FDP} + \mathbf{PYK_2^*} \rightleftharpoons \mathbf{PYK_3^*} \tag{52}$$
$$\text{ADP} + \mathbf{PYK_3^*} \rightleftharpoons \mathbf{PYK_3^*}\text{-ADP} \tag{53}$$
$$\text{PEP} + \mathbf{PYK_3^*} \rightleftharpoons \mathbf{PYK_3^*}\text{-PEP} \tag{54}$$
$$\text{PEP} + \mathbf{PYK_3^*}\text{-ADP} \rightleftharpoons \mathbf{PYK_3^*}\text{-ADP-PEP} \tag{55}$$
$$\text{ADP} + \mathbf{PYK_3^*}\text{-PEP} \rightleftharpoons \mathbf{PYK_3^*}\text{-ADP-PEP} \tag{56}$$
$$\mathbf{PYK_3^*}\text{-ADP-PEP} + \text{H} \rightarrow \text{ATP} + \text{PYR} + \mathbf{PYK_3^*} \tag{57}$$
$$\text{FDP} + \mathbf{PYK_3^*} \rightleftharpoons \mathbf{PYK_4^*} \tag{58}$$
$$\text{ADP} + \mathbf{PYK_4^*} \rightleftharpoons \mathbf{PYK_4^*}\text{-ADP} \tag{59}$$
$$\text{PEP} + \mathbf{PYK_4^*} \rightleftharpoons \mathbf{PYK_4^*}\text{-PEP} \tag{60}$$
$$\text{PEP} + \mathbf{PYK_4^*}\text{-ADP} \rightleftharpoons \mathbf{PYK_4^*}\text{-ADP-PEP} \tag{61}$$
$$\text{ADP} + \mathbf{PYK_4^*}\text{-PEP} \rightleftharpoons \mathbf{PYK_4^*}\text{-ADP-PEP} \tag{62}$$
$$\mathbf{PYK_4^*}\text{-ADP-PEP} + \text{H} \rightarrow \text{ATP} + \text{PYR} + \mathbf{PYK_4^*} \tag{63}$$
$$\mathbf{PYK_0^*} \rightleftharpoons \mathbf{PYK_0^\dagger} \tag{64}$$
$$\text{ATP} + \mathbf{PYK_0^\dagger} \rightleftharpoons \mathbf{PYK_1^\dagger} \tag{65}$$
$$\text{ATP} + \mathbf{PYK_1^\dagger} \rightleftharpoons \mathbf{PYK_2^\dagger} \tag{66}$$
$$\text{ATP} + \mathbf{PYK_2^\dagger} \rightleftharpoons \mathbf{PYK_3^\dagger} \tag{67}$$
$$\text{ATP} + \mathbf{PYK_3^\dagger} \rightleftharpoons \mathbf{PYK_4^\dagger} \tag{68}$$

The active fraction for PYK ($f_\text{A}^\text{PYK}$) is given by the summation of all active forms of the enzyme ($\mathbf{PYK_i^*}$ and bound species) over the total enzyme, $E_\text{T}$, given by:

$$f_\text{A}^\text{PYK} = \frac{\lambda}{\text{E}_\text{T}} \tag{69}$$

where

$$\lambda = \mathbf{PYK_0^*} + \mathbf{PYK_1^*} + \mathbf{PYK_2^*} + \mathbf{PYK_3^*} + \mathbf{PYK_4^*} + \mathbf{PYK_0^*}\text{-PEP} + \mathbf{PYK_1^*}\text{-PEP} + \mathbf{PYK_2^*}\text{-PEP} + \mathbf{PYK_3^*}\text{-PEP} + \mathbf{PYK_4^*}\text{-PEP} + \mathbf{PYK_0^*}\text{-ADP} + \mathbf{PYK_1^*}\text{-ADP} + \mathbf{PYK_2^*}\text{-ADP} + \mathbf{PYK_3^*}\text{-ADP} + \mathbf{PYK_4^*}\text{-ADP} + \mathbf{PYK_0^*}\text{-ADP-PEP} + \mathbf{PYK_1^*}\text{-ADP-PEP} + \mathbf{PYK_2^*}\text{-ADP-PEP} + \mathbf{PYK_3^*}\text{-ADP-PEP} + \mathbf{PYK_4^*}\text{-ADP-PEP}.$$

**Hemoglobin and the Rapoport-Luebering Shunt.** The Rapoport-Luebering (RL) Shunt accounts for the presence of hemoglobin (HB), whose binding to oxygen is regulated by 2,3-diphosphoglycerate (2,3-DPG). We have modeled HB using a cooperative mechanism (i.e., the affinity for oxygen increases with more bound oxygen) with allosteric inhibition by 2,3-DPG (3). We have here used the notation $\mathbf{HB_i^*}$ to denote the relaxed (i.e., active) form $i$ of HB and the notation $\mathbf{HB}^\dagger$ to denote the tense (i.e., inactive) form; $i$ indicates the number of oxygen species bound to HB. The full reaction schema is detailed in Eqs. (70)-(77):

$$\mathbf{HB}^*{}_0 + \text{O}_2 \rightleftharpoons \mathbf{HB}^*{}_1 \tag{70}$$
$$\mathbf{HB}^*{}_1 + \text{O}_2 \rightleftharpoons \mathbf{HB}^*{}_2 \tag{71}$$
$$\mathbf{HB}^*{}_2 + \text{O}_2 \rightleftharpoons \mathbf{HB}^*{}_3 \tag{72}$$
$$\mathbf{HB}^*{}_3 + \text{O}_2 \rightleftharpoons \mathbf{HB}^*{}_4 \tag{73}$$
$$\text{23DPG} + \text{H}_2\text{O} \rightleftharpoons \text{3PG} + \text{P}_\text{i} \tag{74}$$
$$\text{13DPG} \rightleftharpoons \text{23DPG} + \text{H} \tag{75}$$
$$\text{23DPG} + \mathbf{HB}^*{}_0 \rightleftharpoons \mathbf{DHB}^\dagger \tag{76}$$
$$0_2 \rightleftharpoons \varnothing \tag{77}$$

Here, equation 77 represents an exchange reaction with extracellular oxygen. The active fraction for HB ($f_\text{A}^\text{Hb}$) is given by the summation of all active forms of the enzyme over the total enzyme, $E_\text{T}$, given by:

$$f_\text{A}^\text{HB} = \frac{\lambda}{\text{E}_\text{T}} \tag{78}$$

where

$$\lambda = \mathbf{HB}^*{}_0 + \mathbf{HB}^*{}_1 + \mathbf{HB}^*{}_2 + \mathbf{HB}^*{}_3 + \mathbf{HB}^*{}_4.$$

**Disturbance rejection capabilities of models with regulation.** The inclusion of feedback and other regulatory mechanisms are designed to improve the disturbance rejection capabilities of a

system (10). For biological systems, regulatory mechanisms are expected to enable organisms to maintain a robust homeostatic state in the face of environmental perturbations (11, 12). We therefore constructed nine different models representing every combination of enzyme modules (e.g., base model plus PFK, base model plus PFK and HEX). We then (1) investigated the capacity for each of these models to help maintain the homeostatic state and (2) examined how understanding the catalytic potentials help elucidate this ability. We simulated a 50% increase in ATP utilization for 100 hours and calculated the total ATP flux in the network (i.e., total flux through ATP-producing reactions minus total flux through ATP-consuming reactions) for each of the models constructed (Fig A). All systems were able to maintain a stable homeostatic state following the perturbation (Fig A). We calculated the sum of squared error (SSE) for each model in order to quantify the disturbance rejection capabilities of each model (Fig A). As expected, the models with little or no regulation performed the worst, while increased regulation generally lowered the SSE. The base glycolytic model with the PYK module performed the worst, while the model containing the PFK and HEX modules with hemoglobin performed the best. The final steady-state values for the energy charge differed with the inclusion of hemoglobin in the model, although the magnitude of these differences was small (Fig A).

The disturbance rejection capabilities of the models improved with the incorporation of additional regulatory mechanisms (Fig A). We simulated physiologically-relevant perturbations, observing that systems with regulation are improved over those with less regulation (i.e., fewer modules) as shown by quantifying the total deviation of the model output from the setpoint (i.e., the SSE). This increase in robustness with the addition of regulatory reactions corroborate findings from previous studies (13), demonstrating the utility and effectiveness of enzyme modules in capturing the subtle regulatory actions of enzymatic entities. It is notable that models with hemoglobin and either HEX or PFK performed well despite not accounting for all regulatory mechanisms. In particular, the model with PFK, HEX, and HB outperformed the model with all three enzyme modules together; these results indicate that while increased regulation generally improve the system's disturbance rejection capabilities, there is more complicated interplay among the enzyme modules when more than one is present. The most striking result is that the presence of the HB enzyme module (and the RL Shunt) drastically improved the disturbance rejection capabilities of the models. We observe that models that containing the PYK module exhibit lower SSE than models without PYK, likely due to the fact that PYK represents one of the last steps in the model and therefore has a smaller impact on the rest of the system.

## References


1. Santamaria B, Estevez AM, Martinez-Costa OH, Aragon JJ (2002) Creation of an allosteric phosphofructokinase starting with a nonallosteric enzyme. the case of dictyostelium discoideum phosphofructokinase. *J. Biol. Chem.* 277(2):1210–1216.
2. Du B, et al. (2016) Evaluation of rate law approximations in bottom-up kinetic models of metabolism. *BMC Systems Biology* 10(1).
3. Palsson BO (2011) *Systems Biology: Simulation of Dynamic Network States*. (Cambridge University Press, New York).
4. Turányi T, Tomlin AS (2014) *Analysis of Kinetic Reaction Mechanisms*. (Springer Berlin Heidelberg).
5. Monod J, Wyman J, Changeux JP (1965) On the nature of allosteric transitions: A plausible model. *Journal of Molecular Biology* 12(1):88–118.
6. Kosow DP, Rose IA (1970) Product inhibition of the hexokinases. *Journal of Biological Chemistry* 245(1):198–204.
7. Ponce J, Roth S, Harkness DR (1971) Kinetic studies on the inhibition of glycolytic kinases of human erythrocytes by 2, 3-diphosphoglyceric acid. *Biochimica et Biophysica Acta (BBA)-Enzymology* 250(1):63–74.
8. Rijksen G, Staal GE (1976) Purification and some properties of human erythrocyte hexokinase. *Biochimica et Biophysica Acta (BBA) - Enzymology* 445(2):330–341.
9. Valentini G, et al. (2000) The allosteric regulation of pyruvate kinase. *Journal of Biological Chemistry* 275(24):18145–18152.
10. Zames G (1981) Feedback and optimal sensitivity: Model reference transformations, multiplicative seminorms, and approximate inverses. *IEEE Transactions on Automatic Control* 26(2):301–320.
11. Grimbs S, Selbig J, Bulik S, Holzhütter HG, Steuer R (2007) The stability and robustness of metabolic states: identifying stabilizing sites in metabolic networks. *Mol. Syst. Biol.* 3:146.
12. Chakrabarti A, Miskovic L, Soh KC, Hatzimanikatis V (2013) Towards kinetic modeling of genome-scale metabolic networks without sacrificing stoichiometric, thermodynamic and physiological constraints. *Biotechnol. J.* 8(9):1043–1057.
13. Millard P, Smallbone K, Mendes P (2017) Metabolic regulation is sufficient for global and robust coordination of glucose uptake, catabolism, energy production and growth in escherichia coli. *PLOS Computational Biology* 13(2):e1005396.

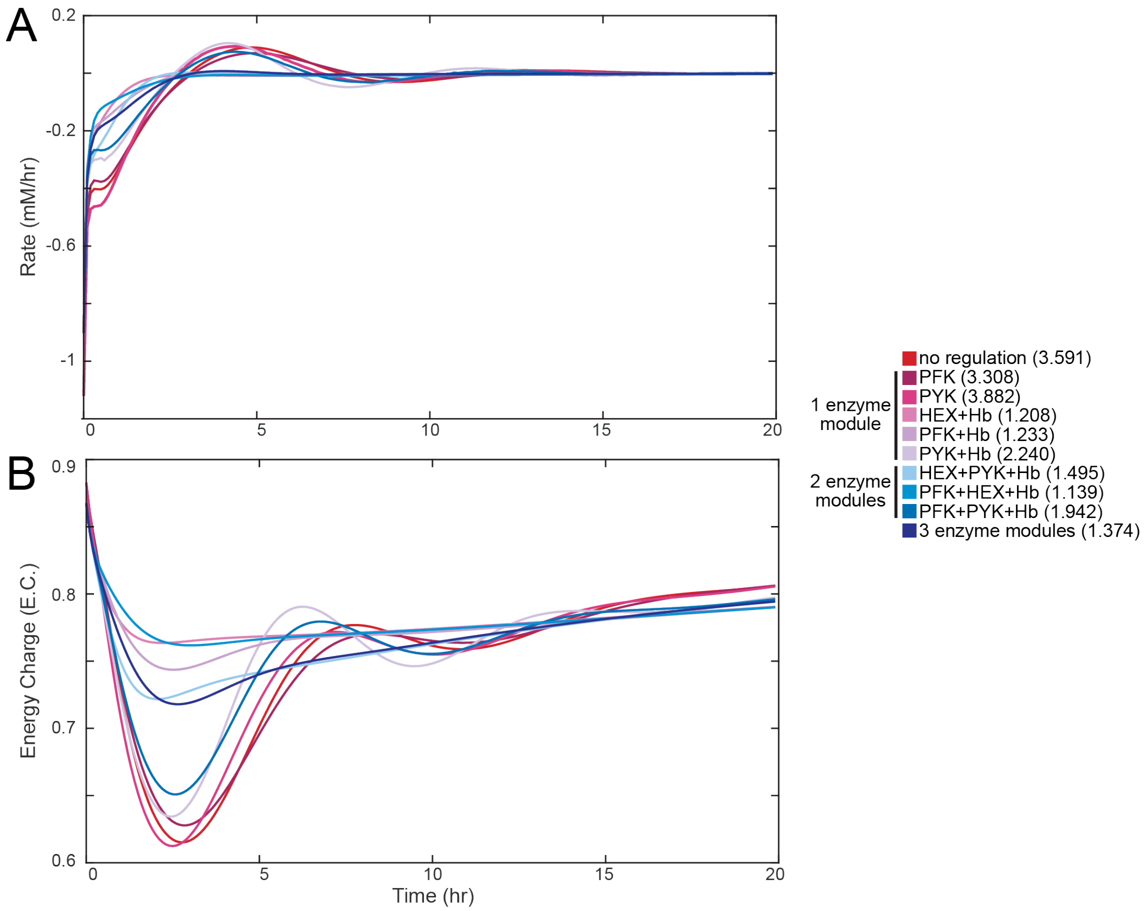


**Fig A.** Reaction rates are not as sensitive to network-level perturbations as systemic variables like the adenylate energy charge. (A) The net rate of ATP usage (i.e., total flux through ATP-producing reactions minus total flux through ATP-consuming reactions) is shown as a function of time. The number in parentheses represents the SSE for each model, quantifying the total deviation of the output from the setpoint. (B) The energy charge is shown as a function of time.

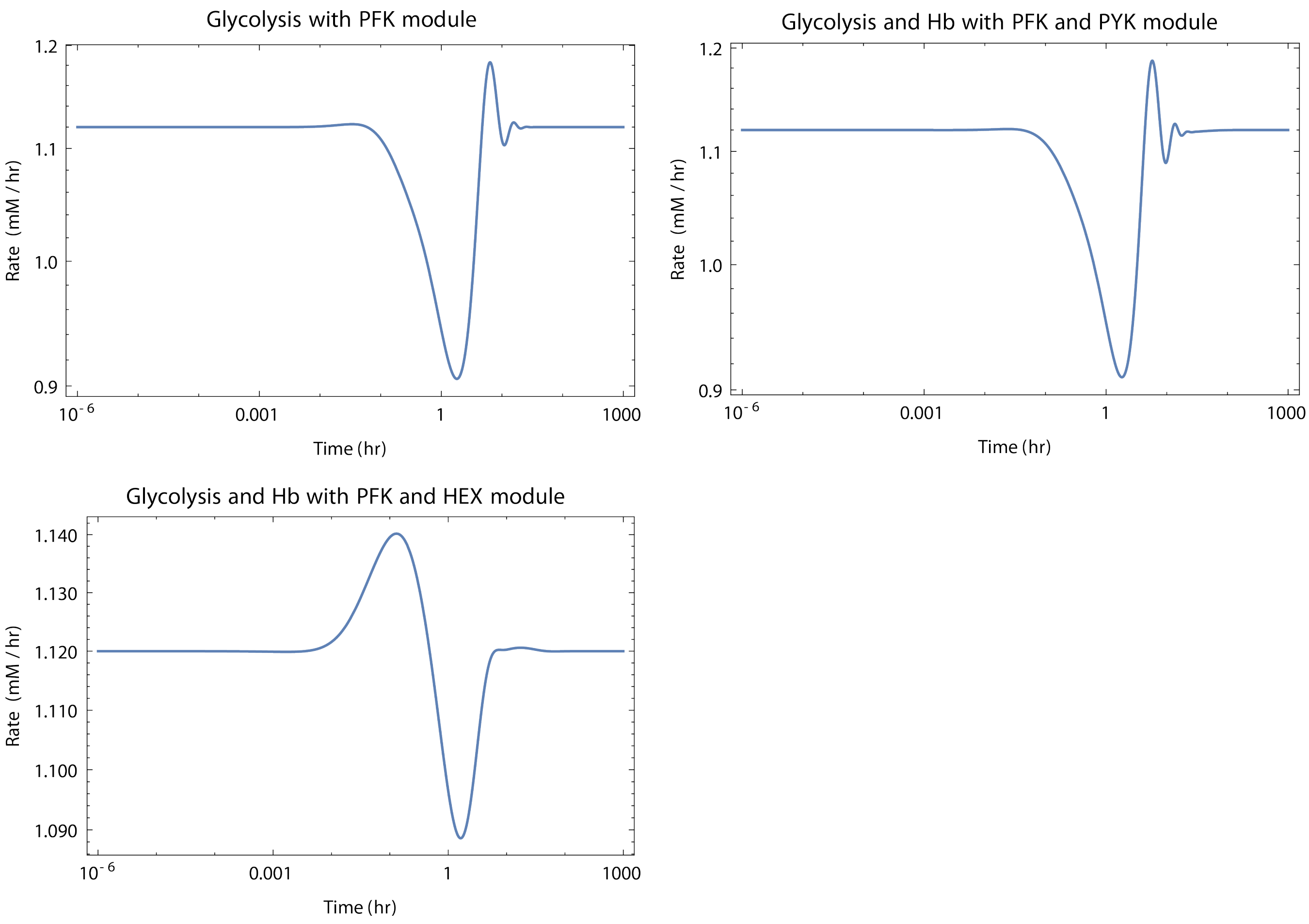


**Fig B.** Rate profiles for three individual models. Rate profiles are shown for PFK for models containing PFK, PFK and PYK with hemoglobin, and PFK and HEX with hemoglobin.

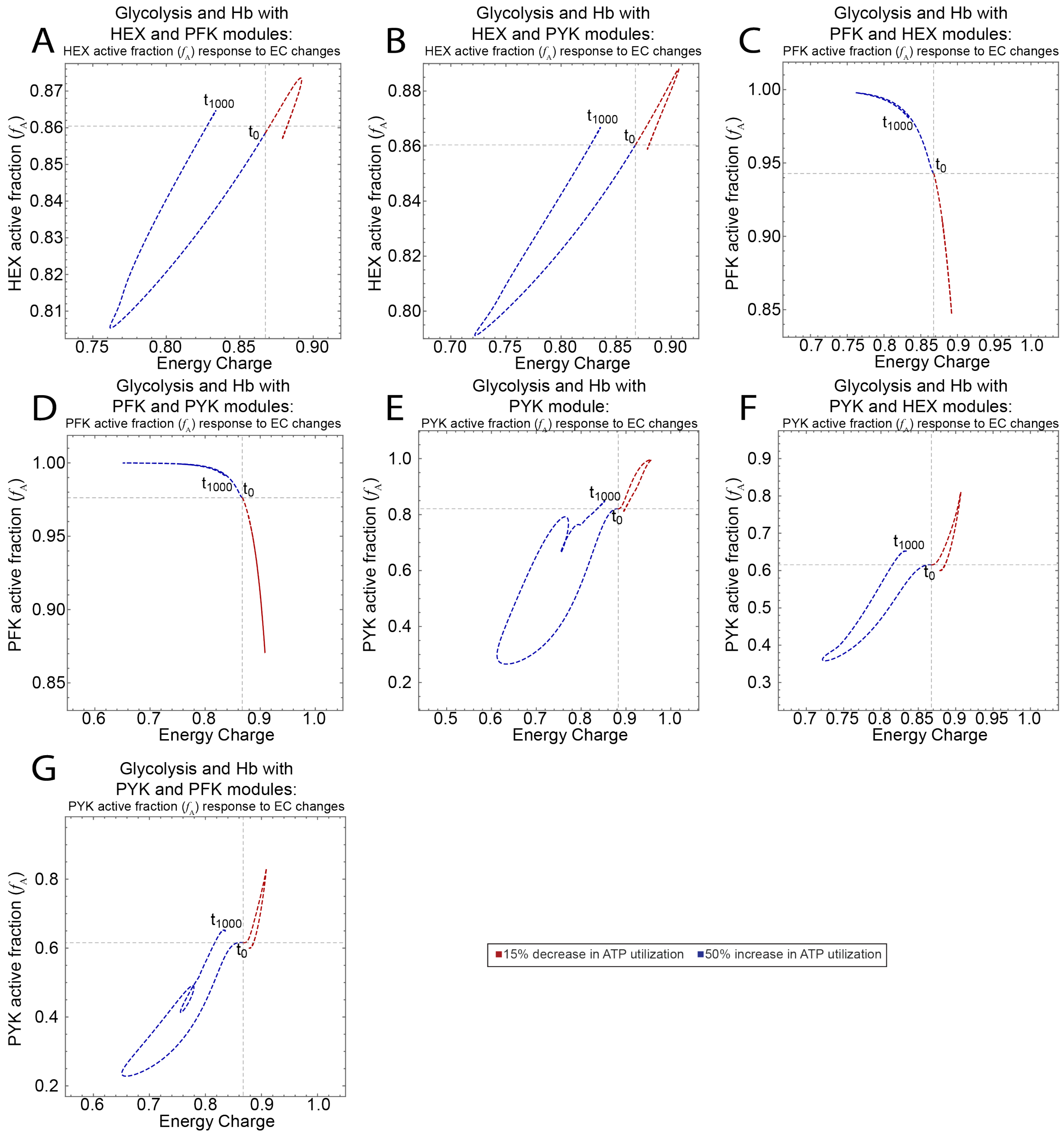


**Fig C.** Catalytic potential plots for all models. (A) glycolysis with HEX and PFK, (B) glycolysis with HEX and PYK, (C) glycolysis with PFK and HEX, (D) glycolysis with PFK and PYK, (E) glycolysis with PYK, (F) glycolysis with PYK and HEX, and (G) glycolysis with PYK and PFK.

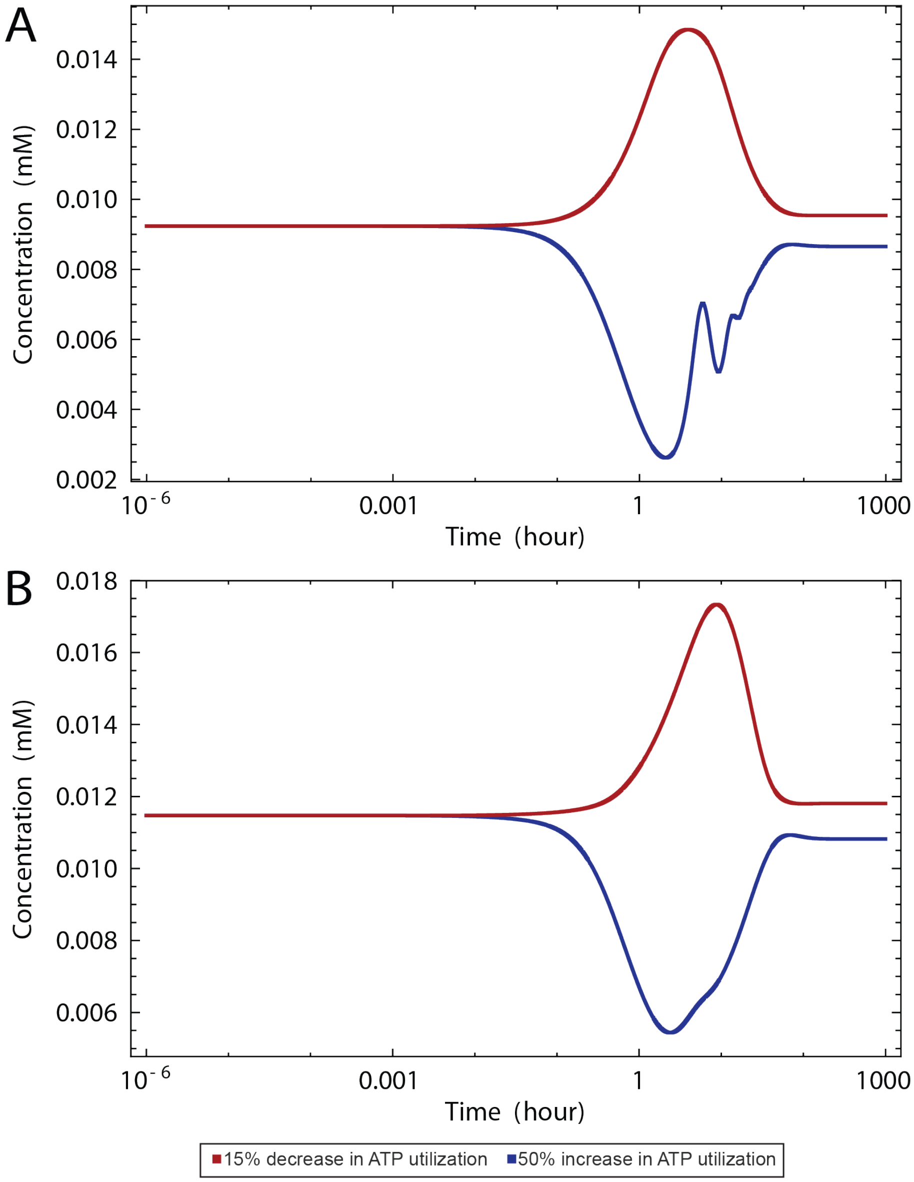


**Fig D.** Concentration plots of fructose 1,6-diphosphate (FDP). (A) Base model (glycolysis, RL Shunt, and hemoglobin) with PYK module. (B) Base model with all three enzyme modules (HEX, PFK, and PYK).

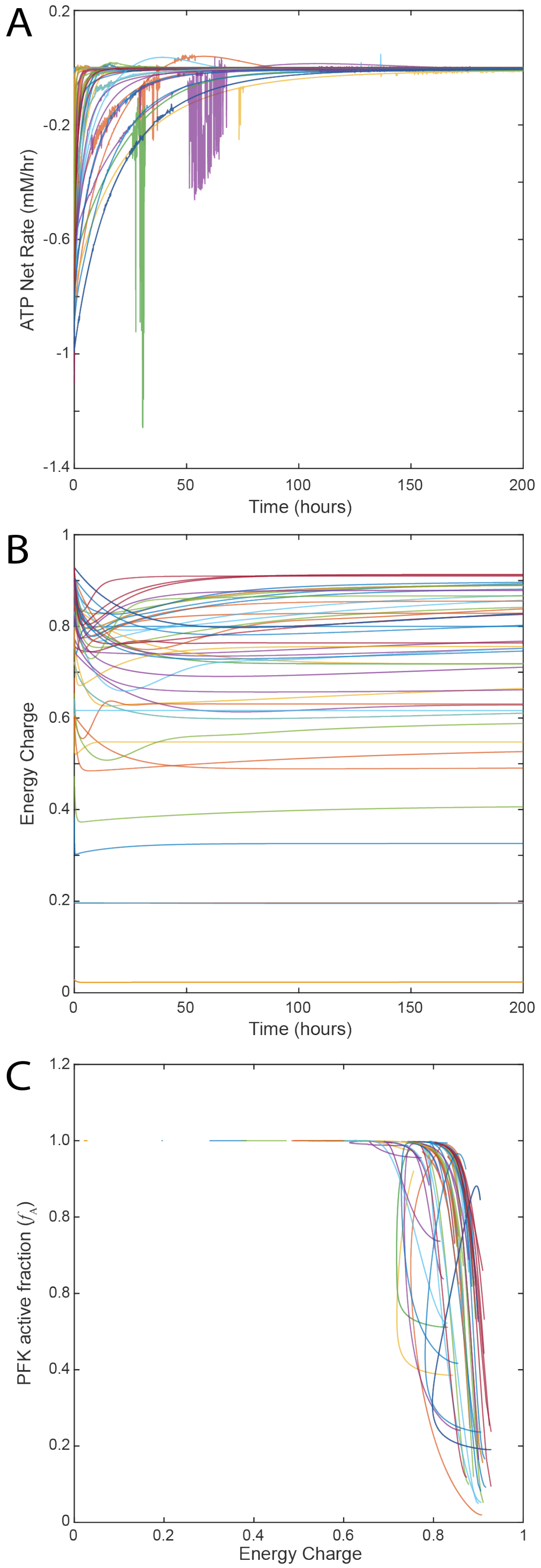


**Fig E.** Sensitivity analysis for 50 randomized models. (A) The net rate of ATP usage (i.e., total flux through ATP-producing reactions minus total flux through ATP-consuming reactions) as a function of time. (B) The energy charge as a function of time. (C) Catalytic potential plots for each randomized model.

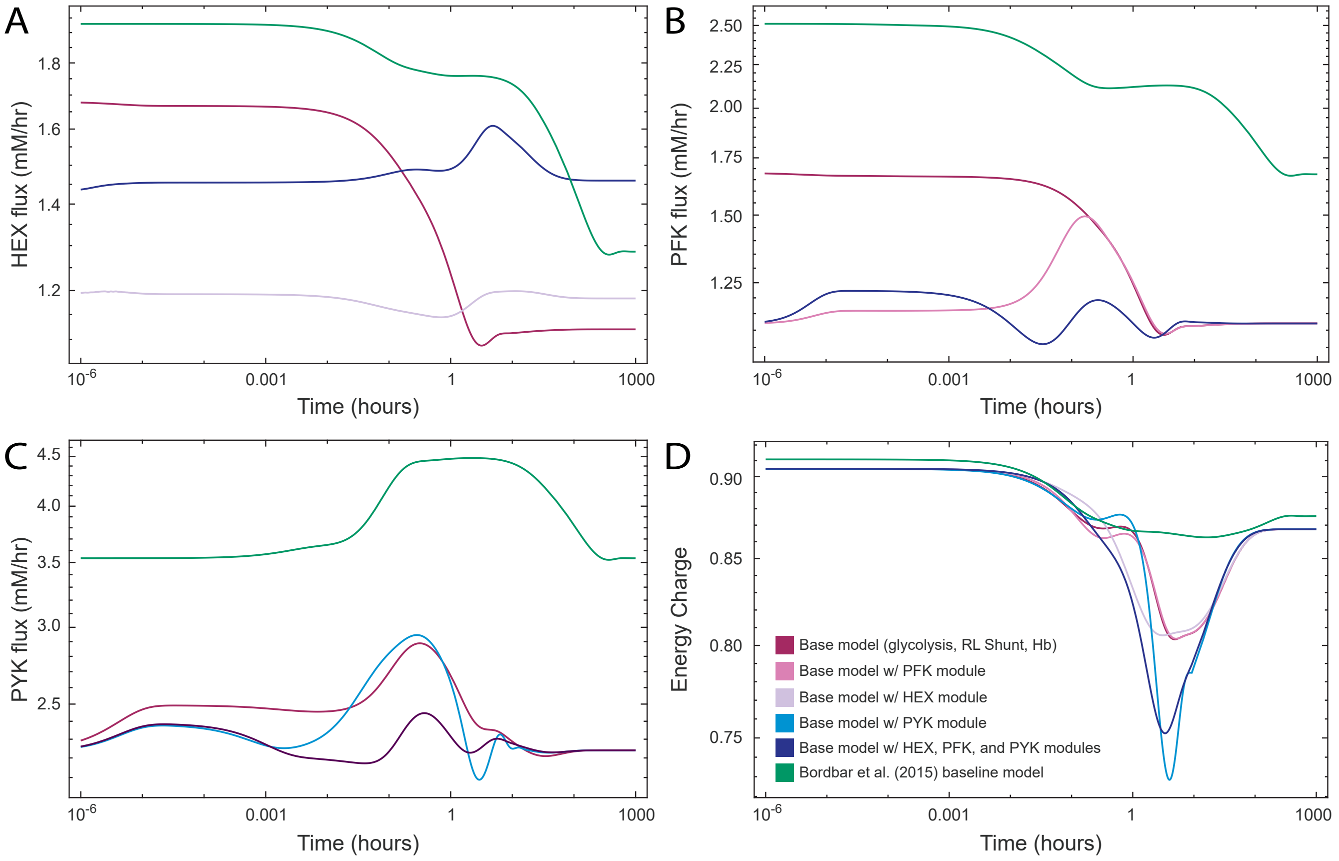


**Fig F.** Comparison against cell-scale model. We show the flux through (A) HEX, (B) PFK, and (C) PYK and also (D) the energy charge as a function of time in response to a pulse that provided a 50% increase in ATP concentration.
